# Detection of carcinogen-induced bladder cancer by fluorocoxib A

**DOI:** 10.1186/s12885-019-6366-x

**Published:** 2019-11-27

**Authors:** Jennifer Bourn, Kusum Rathore, Robert Donnell, Wesley White, Md. Jashim Uddin, Lawrence Marnett, Maria Cekanova

**Affiliations:** 1 Department of Small Animal Clinical Sciences, College of Veterinary Medicine, The University of Tennessee, Knoxville, TN 37996 USA; 2 UT-ORNL Graduate School of Genome Science and Technology, The University of Tennessee, Knoxville, TN 37996 USA; 3 Present address: Department of Cancer Biology, College of Medicine, University of Cincinnati, Cincinnati, OH 45221 USA; 4 Present address: The University of Tennessee Research Foundation, Knoxville, TN 37996 USA; 5 Department of Biomedical and Diagnostic Sciences, College of Veterinary Medicine, The University of Tennessee, Knoxville, TN 37996 USA; 6 Department of Urology, The University of Tennessee, Graduate School of Medicine, Knoxville, TN 37920 USA; 7 A. B. Hancock, Jr., Memorial Laboratory for Cancer Research, Departments of Biochemistry, Chemistry and Pharmacology, Vanderbilt Institute of Chemical Biology, Center for Molecular Toxicology and Vanderbilt-Ingram Cancer Center, Vanderbilt University School of Medicine, Nashville, TN 37232 USA

**Keywords:** Bladder cancer, Optical imaging, Cox-2, Carcinogenesis, Fluorocoxib A

## Abstract

**Background:**

Conventional cystoscopy can detect advanced stages of bladder cancer; however, it has limitations to detect bladder cancer at the early stages. Fluorocoxib A, a rhodamine-conjugated analog of indomethacin, is a novel fluorescent imaging agent that selectively targets cyclooxygenase-2 (COX-2)-expressing cancers.

**Methods:**

In this study, we have used a carcinogen N-butyl-N-4-hydroxybutyl nitrosamine (BBN)-induced bladder cancer immunocompetent mouse B6D2F1 model that resembles human high-grade invasive urothelial carcinoma. We evaluated the ability of fluorocoxib A to detect the progression of carcinogen-induced bladder cancer in mice. Fluorocoxib A uptake by bladder tumors was detected ex vivo using IVIS optical imaging system and Cox-2 expression was confirmed by immunohistochemistry and western blotting analysis. After ex vivo imaging, the progression of bladder carcinogenesis from normal urothelium to hyperplasia, carcinoma-in-situ and carcinoma with increased Ki67 and decreased uroplakin-1A expression was confirmed by histology and immunohistochemistry analysis.

**Results:**

The specific uptake of fluorocoxib A correlated with increased Cox-2 expression in progressing bladder cancer. In conclusion, fluorocoxib A detected the progression of bladder carcinogenesis in a mouse model with selective uptake in Cox-2-expressing bladder hyperplasia, CIS and carcinoma by 4- and 8-fold, respectively, as compared to normal bladder urothelium, where no fluorocoxib A was detected.

**Conclusions:**

Fluorocoxib A is a targeted optical imaging agent that could be applied for the detection of Cox-2 expressing human bladder cancer.

## Background

Bladder cancer is the 6th most common type of cancer with an estimated 80,000 newly diagnosed cases and 17,000 deaths per year in the United States [1]. Bladder cancer incidence is four times higher in men than in women. The most common type of bladder cancer is urothelial carcinoma, also known as transitional cell carcinoma, which accounts for over 90% of all bladder cancer cases in the United States. The extent of the bladder cancer spread through the body is determined by a staging based on physical exams, biopsies, surgery, and imaging tests. The staging system in TNM system is the most often used for the bladder cancer. In that staging system T indicates the spread of tumor through the bladder wall and nearby tissues, N indicates any cancer has spread to lymph nodes near the bladder, and M indicates any cancer has spread (metastasized) to distal sites and organs. There are five stages of bladder cancer, with stage IV being the most advanced metastatic disease stage. The cancers at Stage 0a (Ta, N0, M0) found on the surface of the inner lining of the bladder, Stage 0is (Tis, N0, M0) classified as a flat tumor or carcinoma-in-situ (CIS) and Stage I (T1, N0, M0) belong to a group of non-muscle invasive bladder carcinomas (NMIBC). The cancers at Stages II, (T2a or T2b, N0, M0), Stage IIIA (T3a, T3b or T4a, N0, M0; or T1-4a, N1, M0), Stage IIIb (T1-4a, N2 or N3, M0), Stage IVA (T4b, Any N, M0 or Any T, Any N, M1a) and Stage IVB (Any T, Any N, M1b) are more advanced stages, as the cancer has progressed through the muscle layer of the bladder wall to surrounding local pelvic and later to distal organs, such as bones, liver or lungs (M1b) belong to muscle-invasive bladder cancers (MIBC) [2].

Treatment management depends on whether the bladder cancer is diagnosed as NMIBC or MIBC. Currently, the gold-standard treatment for MIBC is neoadjuvant platinum-based chemotherapy followed by radical cystectomy [3, 4]. In an attempt to reduce the morbidity associated with open radical cystectomy, less invasive approaches, such as laparoscopic/robotic cystectomy have been explored [5, 6]. Standard treatments for NMIBC are a transurethral resection of bladder tumor (TURBT) or opened radical cystectomy depending on patient preferences and anatomy and location of cancer. Risk stratification based on accurate pathologic staging is then employed to determine the need for adjuvant intravesical treatment with chemotherapy (mitomycin C or gemcitabine) or immunotherapy (Bacille Calmette-Guérin) [7–9]. The detection of bladder cancer at the early stages and more accurate detection of cancer during TURBT procedures is needed to improve patient treatment outcomes.

White light cystoscopy (WLC) is the current standard of care for the detection of papillary or larger cancerous lesions in the bladder. WLC has been used for several decades to detect bladder tumors, but there are several limitations associated with WLC, including difficulties in detecting early non-invasive stages of bladder cancer (Ta, T1, CIS), as well as the inability to detect tumor margins during resection procedures leading to the potential for incomplete resection of the tumor [10]. Newer technologies, including fluorescence cystoscopy/photodynamic diagnosis (PDD), narrow band imaging (NBI), confocal laser endomicroscopy (CLE), and optical coherence tomography (OCT) [11, 12] have been developed to improve the quality of detection of the non-invasive disease from MIBC lesions during diagnostic and resection procedures [13, 14]. Fluorescence cystoscopy/PDD and NBI better visualize the tumors and optimize detection of early non-invasive stages of bladder cancer. On the contrary, CLE and OCT further characterize the detected lesions to improve accuracy in determining the grade and stage of the lesions. Fluorescent cystoscopy requires the administration of a contrast agent, which selectively binds to the cancer cells to improve visualization and differentiation of the cancer from normal tissue during resection procedures [15]. Photodynamic diagnosis/blue-light cystoscopy (BLC) is an FDA-approved procedure, which requires the intravesical administration of 5-aminolevulinic acid (5-ALA) or hexaminolevulinate (HAL) directly into the bladder [16–19]. The dye is absorbed by the bladder tissue and after excitation by a light, it emits a red color allowing the better visualization of the tumor during the cystoscopy procedure. Previous studies indicates that BLC can detect bladder tumors more effectively than WLC, at both early and late stages [18–22] and is now recommended as standard of care when available.

Cyclooxygenase-2 (Cox-2) is aberrantly expressed in bladder cancer and is one of the key proteins responsible for angiogenesis [23, 24] and tumorigenesis [25, 26]. The increased Cox-2 expression has also been reported to be correlated with tumor grade and poor clinical outcome for patients diagnosed with bladder cancer [27–30]. The overexpression of Cox-2 in bladder cancer tissue can be used as a biomarker for the detection of bladder cancer and as a prognostic marker for outcome. Fluorescently labeled Cox-2 inhibitors used for targeted optical imaging could assist for the early detection of non-invasive disease before it metastasized. Fluorocoxib A is a rhodamine-conjugated analog of indomethacin that selectively targets Cox-2 in solid tumors [31]. Fluorocoxib A has been validated previously for the detection of LPS-induced inflammation in a rat model [31] and in Cox-2-expressing cancers in vitro [32] and in vivo [33, 34].

There are several models currently available for the study of bladder carcinogenesis, including genetically or carcinogens induced tumors in the rodents [35, 36]. In our study, we used a well-established BBN-induced mice bladder cancer model. BBN belongs to nitrosamines that is a highly carcinogenic group of compounds [37] known to induce hepatic, gastric, and bladder cancers [38, 39]. BBN is administered orally either in drinking water or by oral gavage at doses that range from 0.01–0.05% [40].

In this study, we evaluated fluorocoxib A for detection of the Cox-2-expressing, carcinogen-induced bladder cancer in immunocompetent B6D2F1 mice. We validated the specificity of fluorocoxib A to detect both the early in addition to late stages of bladder cancer in vivo*.*

## Methods

### Antibodies and reagents

The antibodies for uroplakin-1a (UP-1a, C-18, sc-15,173) and actin (C-11, sc-1615) were purchased from Santa Cruz Biotechnology (Santa Cruz, CA); antibody for Ki67 (SP6, ab16667) was purchased from Abcam Inc. (Cambridge, MA); antibody for Cox-2 (aa 570–598, 160106) was purchased from Cayman Chemical (Ann Arbor, MI); and secondary anti-rabbit antibody was obtained from Cell Signaling Technology (Danvers, MA). A carcinogen, N-butyl-N-(4-hydroxybutyl) nitrosamine (BBN) was obtained from Sigma-Aldrich (St. Louis, MO). Fluorocoxib A, a *N*-[(5-carboxy-X-rhodaminyl) but-4-yl]-2-[1-(4-chlorobenzoyl)-5-methoxy-2-methyl-1*H*-indol-3-yl] acetamide was synthesized as described [31]. All other chemicals and reagents were purchased from Thermo Fisher Scientific (Pittsburgh, PA), unless otherwise specified.

### Animals

All animal experiments were performed in accordance with approved the University of Tennessee Institutional Animal Care and Use Committee (IACUC) protocol#1892 and in an accordance with all federal, and state guidelines, policies, and regulations to protect animal welfare. The University of Tennessee policies for animal care and use encompass regulations of the Animal Welfare Act as amended (Public Law 99–198 – The Improved Standard for Laboratory Animals Act), Guide for the Care and Use of Laboratory Animals (8th Ed.) and The Guide for the Care and Use of Agricultural Animals in Research and Teaching. The University of Tennessee IACUC is accredited by the Association for Assessment and Accreditation of Laboratory Animal Care (AAALAC). Thirty 5-wk old B6D2F1 female mice (Taconic, Hudson, NY) were randomly divided into three groups (*n* = 10/group). Mice were housed at UT IACUC approved satellite facility for rodents in large standard cages of ten mice per cage in a 12 h /12 h light/dark cycle, with mean temperature of 23 ± 2 °C and relative humidity of 55 ± 10%. Mice were fed with access to standard chow and water ad libitum. Mice in Group 1 served as the control and received only tap drinking water for 18 weeks (Group 1 – 18wks H_2_O). Mice in the other two groups were exposed to BBN for 12 weeks (Group 2 – 12wks BBN) and 18 weeks (Group 3 – 18wks BBN). BBN was administered ad libitum at 0.05% in drinking water to mice. Body weight of each mouse and water consumption of mice per each group was recorded weekly. No adverse events connected with the administration of BBN were detected in mice during duration of our study.

### Optical imaging

Mice were injected with fluorocoxib A (1 mg/kg, s.c.) after the treatment with BBN at 12 and 18 weeks, respectively, and specific fluorocoxib A uptake was detected 4 h post-injection by the Xenogen IVIS Lumina optical imaging system. After mice were euthanized using anesthetic overdose of inhaled isoflurane until complete stopped breathing and followed by a blood withdraw through cardiac left ventricle stick, the tissues were dissected, photographed, and imaged by IVIS system ex vivo (DsRed filters with excitation 500–550 nm, emission 575–650 nm, and background 460–490 nm, 1 s, binning factor 4). The obtained total radiant efficiency [p/s]/[μW/cm^2^] of labeled regions of interest of dissected bladder and other tissues (blood, kidney, liver, lung, heart, muscle, spleen, pancreas, and fat) were evaluated. The values of total radiant efficiency of the bladder were normalized to blood and reported as Tumor-to-Noise Ratio (TNR) values for fluorocoxib A uptake in bladder. After imaging, the dissected bladder was divided into smaller pieces for further analysis. A piece of bladder tissue was fixed in 10% neutral buffered formalin for histology and immunohistochemistry (IHC) analysis. Another piece of bladder was kept in RNA*later* solution and stored at − 80 °C until Western blotting (WB) analyses were performed.

### Histology

Dissected tissue samples from mice were formalin-fixed paraffin-embedded and sectioned at 7 μm. Hematoxylin and eosin (H & E) staining was performed following standard protocol by the histology service of the University of Tennessee Veterinary Medical Center in Knoxville. The group assignment of mice bladders tissue sections was blinded to a board-certified veterinary pathologist (RD) for the objective histological evaluation and scoring to determine the progression of BBN-induced carcinogenesis. The histological analysis of the H & E sections of the bladder tissue from each mouse was recorded to quantify the prevalence of BBN-induced inflammation, hyperplasia, CIS, and carcinoma among the experimental groups according to scoring system as mentioned in the Table 1. The scoring and type definition of histological evaluation of inflammation (characterized by the presence of specific immune cells lymphocytes, macrophages, neutrophils, and plasma cells), hyperplasia, carcinoma-in-situ (CIS), and carcinoma was summarized in the Table 1. CIS in a mouse BBN-induced urothelial carcinoma model was defined as a carcinoma confined to the urothelium where the malignant urothelial (transitional) cells have loss of cell polarity, present cellular atypia, have increased number of mitotic figures, and large irregular nuclei with a high nuclear to cytoplasmic ratio (adapted from Stanford medicine surgical pathology criteria).

**Table 1:** Description of scoring summary used for the histology evaluation of bladder from mice

	Score/Type	Description
Inflammation	0	no presence of inflammatory cells
	1	low presence of inflammatory cells
	2	moderate presence of inflammatory cells
	3	high presence of inflammatory cells
	N, L, P, M	Type of inflammatory cells - Neutrophils, Lymphocytes, Plasma cells, Macrophages
Hyperplasia	0	no hyperplasia (less than 2 cells in urothelial layer) present
	1	low hyperplasia (between 3 and 5 cells in urothelial layer) present
	2	severe hyperplasia (more than 5 cells in urothelial layer) present
	D, F, M	Diffuse, Focal, Multi-focal hyperplasia
Carcinoma in situ (CIS)	0	no CIS present
	Yes	CIS present
Carcinoma^a^	0	no carcinoma present
	Yes	carcinoma present

Notes: ^a^Including adenocarcinoma, squamous cell carcinoma and transitional cell carcinoma

### Immunohistochemistry (IHC)

The IHC staining was performed as described previously [34]. After de-paraffinization of tissue sections, the antigen retrieval using sodium citrate pH 6.0 was performed for 20 min in the antigen retriever (Electron Microscopy Sciences, Hatfield, PA). Blocking of endogenous peroxidase activity was performed using hydrogen peroxide, tissues were incubated with primary antibodies (Ki67, UP-1a, and Cox-2) followed by the incubation with the biotinylated secondary antibodies, followed by streptavidin/HRP detection system, and visualized by 3,3′-diaminobenzidine (DAB) staining. Nuclei were counter-stained with hematoxylin and slides were evaluated using a Leitz DMRB microscope (Leica). The images were captured by a DP73 camera (Hunt Optics and Imaging, Pittsburgh, PA) using CellSens Standard software (Olympus, Center Valley, PA).

### Western blotting (WB)

The WB was performed according to standard WB protocol as described previously [34]. Briefly, the tissue samples were lysed on an ice-cold RIPA buffer supplemented with a protease and phosphatase inhibitors cocktail and briefly sonicated on ice. Protein concentrations were measured using Pierce® BCA protein assay (Thermo Scientific, Rockford, IL). Equal amounts of proteins were loaded onto SDS-PAGE gels and transferred into nitrocellulose membranes. After blocking, the membranes were incubated with primary antibodies overnight at 4 °C followed by incubation with horseradish peroxidase-conjugated secondary antibodies for 1 h at room temperature. The immuno-reactive bands were visualized using the ECL prime chemiluminescence system (GE Healthcare Life Sciences, Marlborough, MA) and the images were captured using the BioSpectrum® 815 imaging system (Analytik Jena, Upland, CA). Densitometry analysis was performed using the VisionWorks® acquisition and analysis software (Analytic Jena).

### Statistical analysis

Statistical analysis was conducted using the paired Student’s *t-*test to establish the significant differences among treatment groups. Results were considered statistically significant at *p < 0.05, **p < 0.01, and ***p < 0.001.

## Results

### Fluorocoxib A uptake by BBN-induced bladder cancer

**Fig. 1:**
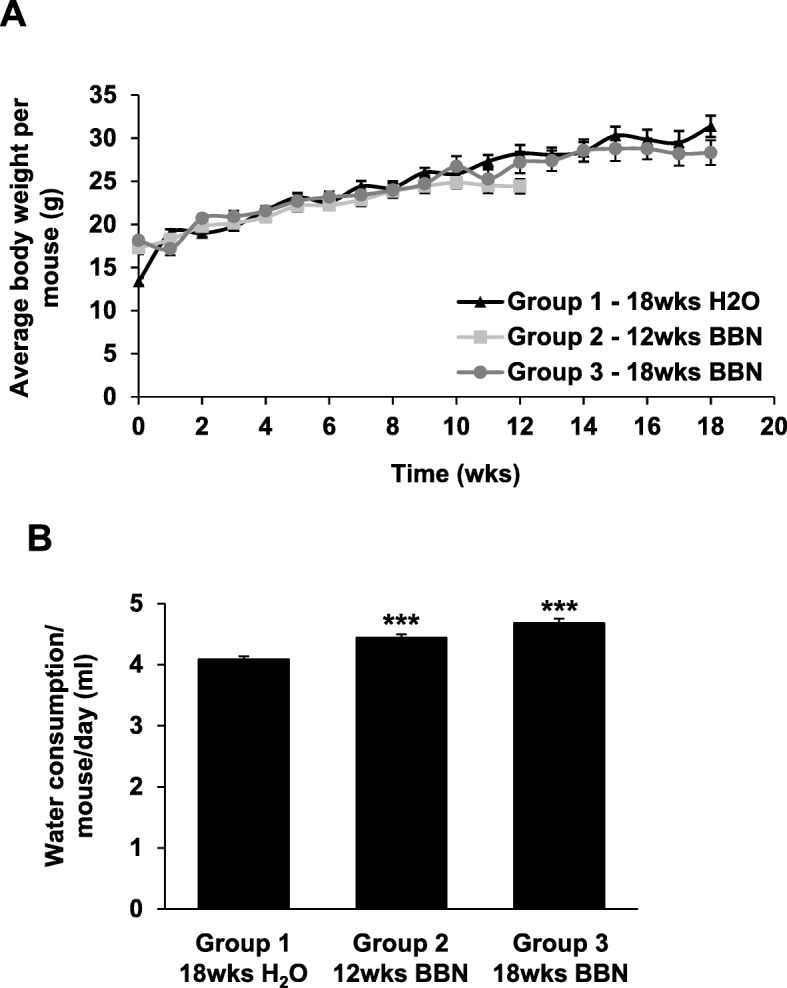
BBN-induced bladder cancer mouse model. **a** Female B6D2F1 mice were exposed to 0.05% BBN *ad libidum* in drinking water for 12 weeks (*n* = 10; Group 2 – 12wks BBN) and 18 weeks (n = 10; Group 3 – 18wks BBN). Mice without BBN treatment for 18 weeks (*n* = 10; Group 1 – 18wks H_2_O) served as a control. Body weight (g) of mice was recorded weekly. No effects of BBN on a body weight of mice was detected as compared to control mice. **b** A slightly increased daily water consumption per mouse was observed in mice from Group 2 – 12wks BBN and Group 3 – 18wks BBN when compared to the mice from control Group 1 – 18wks H_2_O. Data show mean ± SE of the daily drinking water consumption (ml) per mouse from each group (*n* = 10). Significance between BBN and control groups was assessed using a two-tailed paired Student’s *t*-test (***p < 0.001)

BBN treatment had no adverse effect on the growth of the mice over time as no remarkable differences in the body weight of mice were observed between groups as shown in Fig. 1a. A small increase in averaged daily water consumption was observed in mice from Group 2 – 12wks BBN and Group 3 – 18wks BBN (***p < 0.001) when compared to mice from Group 1 - 18wks H_2_O as shown in Fig. 1b.

**Fig. 2:**
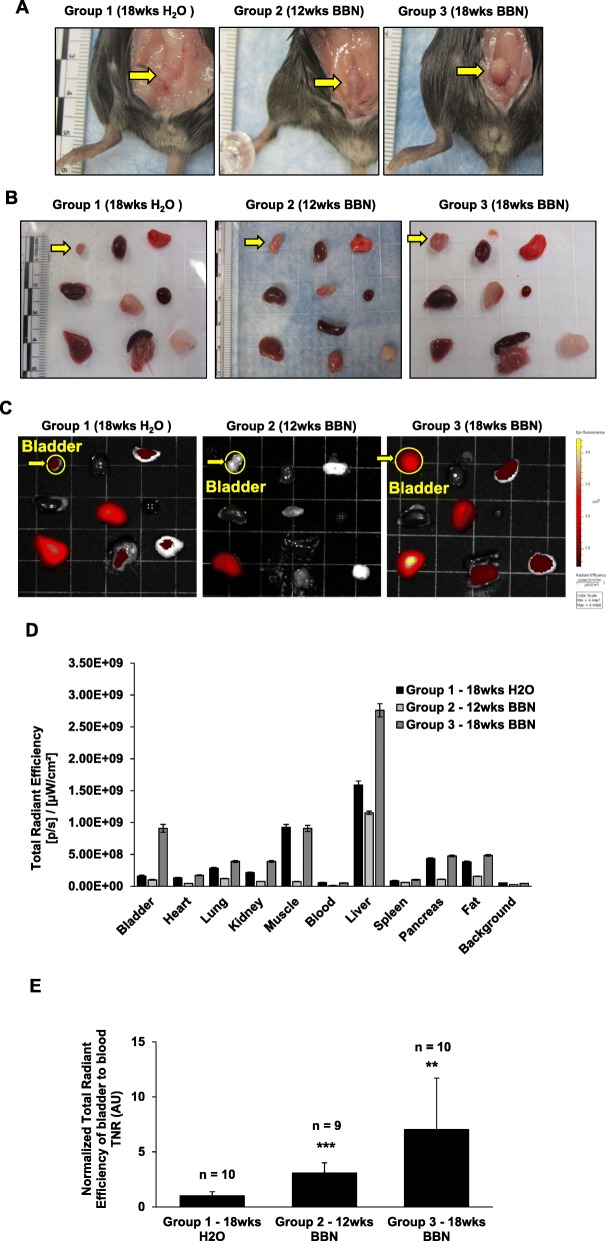
Fluorocoxib A uptake by BBN-induced bladder cancer in mice. **a** After BBN treatment, fluorocoxib A (1 mg/kg, s.c.) was administrated in mice followed by the IVIS imaging. Four hours after fluorocoxib A administration, mice were sacrificed, and dissected tissues were imaged by the IVIS Lumina imaging system. The representative photographs of empty bladders (yellow arrow) from mice from each treatment group. **b** and **c** The representative photographs and IVIS ex vivo images of dissected organs of mice*.* Organs from left to right: Row 1 – bladder (yellow arrow pointed to yellow circle), heart, lung; Row 2 - kidney, muscle, blood; Row 3 - liver, pancreas & spleen, fat. Higher uptake of fluorocoxib A was observed in mice bladder, liver and muscle tissues. **d** Total radiant efficiency ([p/s]/[μW/cm^2^]) values of dissected organs from mice from each group (*n* = 10, *n* = 9, *n* = 10). Data show mean ± S.E. of the total radiant efficiency values of individual organs from mice from each group (*n* = 10, *n* = 9, *n* = 10). **e** Normalized total radiant efficiency values of fluorocoxib A uptake in bladders to blood (TNR). The 3- and 7-fold increase in fluorocoxib A uptake was detected in bladders dissected from mice in Group 2 – 12wks BBN and Group 3 – 18wks BBN (***p < 0.001, **p < 0.01), respectively, compared to the dissected bladder from control mice. Data show mean ± S.E. of the normalized total radiant efficiency values of bladder from mice per each group (*n* = 10, *n* = 9, *n* = 10). Significance in fluorocoxib A uptake by bladder of mice from BBN and control groups was assessed using a two-tailed paired Student’s *t*-test (**p < 0.01, ***p < 0.001)

To detect the BBN-induced bladder cancer, fluorocoxib A was administered (1 mg/kg, s.c.) to mice at the end of BBN exposure for 12wks and 18wks and imaged by the IVIS imaging system. Mice from the control group (Group 1 – 18wks H_2_O) were imaged at the same time as mice from Group 3 - 18wks BBN. Four hours after fluorocoxib A administration, mice were sacrificed, and dissected tissues were imaged by the IVIS imaging system to detect fluorocoxib A uptake. The empty bladders of mice in Group 2 – 12wks BBN and Group 3 – 18wks BBN were larger when compared to bladders of mice from control group (Group 1 – 18wks H_2_O) as shown in Fig. 2a and b (yellow arrow). No other abnormal gross pathological changes of other organs, including heart, lung, kidney, liver, pancreas, and spleen were observed during necropsy (performed by MC) as shown in Fig. 2b. Fluorocoxib A uptake was detected primarily in bladder, however, also in liver and muscle tissues as shown in Fig. 2c and d. Total radiant efficiency values of bladders were normalized to blood (TNR) and significant 3- and 7-fold increases in fluorocoxib A uptake by bladders from mice in Group 2 – 12wks BBN and Group 3 – 18wks BBN, respectively, (***p < 0.001, **p < 0.01, respectively) compared to bladders from untreated mice (Group 1 – 18wks H_2_O) were detected as shown in Fig. 2d and e.

### Progression of bladder carcinogenesis by BBN

**Fig. 3:**
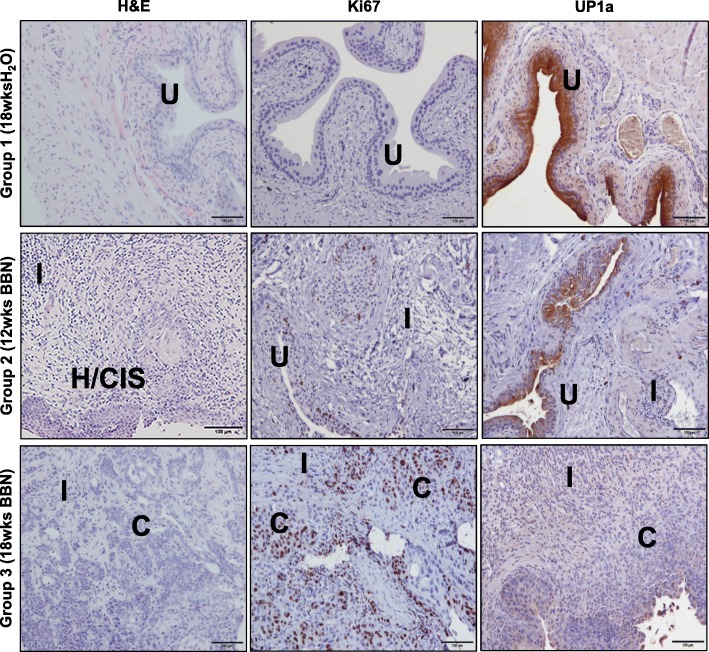
Progression of bladder cancer by BBN. The BBN-induced bladder cancer progression from normal urothelium to hyperplasia/CIS and to invasive carcinoma with increased inflammation in bladder of mice was confirmed by H & E staining (left panels). The progression of bladder carcinogenesis was confirmed by increased expression of Ki67 positive urothelial cells in bladder from mice exposed to BBN (middle panels). In addition, uroplakin-1A, a protein that is highly expressed in normal bladder urothelium was downregulated in BBN-induced bladder cancers as confirmed by IHC analysis (right panels). Images were taken by Leitz DMRB microscope, scale bar 100 μm. C – carcinoma; CIS – carcinoma in situ; H – hyperplasia; I - inflammation; U - normal urothelium

The histopathology of the dissected bladder tissues was assessed by H & E and IHC staining for detection of Ki67 and uroplakin-1A (UP1a) expression (Fig. 3). The BBN-induced bladder cancer progression from normal urothelium to hyperplasia and to invasive carcinoma with presence of intensive inflammation in bladder of the mice as confirmed by H & E staining (Fig. 3, left panels). The histological analysis of the H & E sections of the bladder tissue from each mouse was recorded to quantify the prevalence of BBN-induced inflammation, hyperplasia, CIS, and carcinoma lesions among the experimental groups according to scoring system as mentioned in the Table 1. As shown in Table 2, mice from Group 2 – 12wks BBN and Group 3 - 18wks BBN had increased incidence of inflammation, hyperplasia, and bladder carcinoma lesions when compared to mice from Group 1 – 18wks H_2_O (control). The progression of bladder carcinogenesis by BBN was also confirmed by the presence of the increased number of bladder tumor and carcinomas cells with positive Ki67 expression in nuclei (Fig. 3, middle panels). In addition, UP1a, a protein that is highly expressed in normal bladder urothelium, was downregulated by BBN-induced bladder hyperplasia/CIS and carcinoma lesions (Fig. 3, right panels).

**Table 2:** Histology evaluation of bladder tissues from individual mice among treatment groups with normalized total radiant efficiency values of bladder to blood by IVIS detection system

	Mouse #	Inflammation (Infl)	Hyperplasia (Hyp)	Carcinoma in situ (CIS)	Carcinoma (CA)	Normalized Total Radiant Efficiency of bladder to blood by IVIS
Group 1 18wks H_2_o	1–1	1 L	0	0	0	1.50
	1–2	0	1 F	0	0	2.36
	1–3	3 LF	1 F	0	0	5.47
	1–4	0	0	0	0	2.53
	1–5	0	0	0	0	2.72
	1–6	0	0	0	0	3.55
	1–7	0	0	0	0	1.88
	1–8	n/a	n/a	n/a	n/a	2.89
	1–9	n/a	n/a	n/a	n/a	3.46
	1–10	0	0	0	0	2.89
Group 2 12wks BBN	2–1^a^	n/a	n/a	n/a	n/a	n/a
	2–2	2 NPL 1 M	0	0	0	7.11
	2–3	1 NL	1 M	0	0	10.50
	2–4	3 LP 1 M	no urothelium	no urothelium	no urothelium	11.68
	2–5	3 LP	no urothelium	no urothelium	no urothelium	8.82
	2–6	2 NPL rareM	2 L	Yes	Yes	6.64
	2–7	2 LP 1 M	1 M	0	0	12.93
	2–8	1 L	minimal urothelium	0	0	10.62
	2–9	1 NPL 1 M	1 M	0	0	4.27
	2–10	1 NLP 2 M	1 M	Yes	Yes	8.31
Group 3 18wks BBN	3–1	1 LPNM	1 D	Yes	0	7.81
	3–2	3 PNL 1 M	2 M	Yes	Yes	35.41
	3–3	2 LP 1 M	2	0	0	24.88
	3–4	3 LN 1 M	2	Yes	0	5.84
	3–5	2 LM	1	0	Yes	47.67
	3–6	2 LM	1	0	Yes	5.77
	3–7	2 LNM	1	0	Yes	25.67
	3–8	2 LNM	2	0	Yes	15.18
	3–9	3 LNPM	1	0	Yes	23.81
	3–10	2 LN 1 M	1 D	0	0	13.43

Notes: n/a - No bladder tissue was available for histology evaluation; ^a^Mouse#2–1 died before the end of experiment with unknown cause of death

### Upregulation of Cox-2 by BBN in bladder carcinoma

**Fig. 4:**
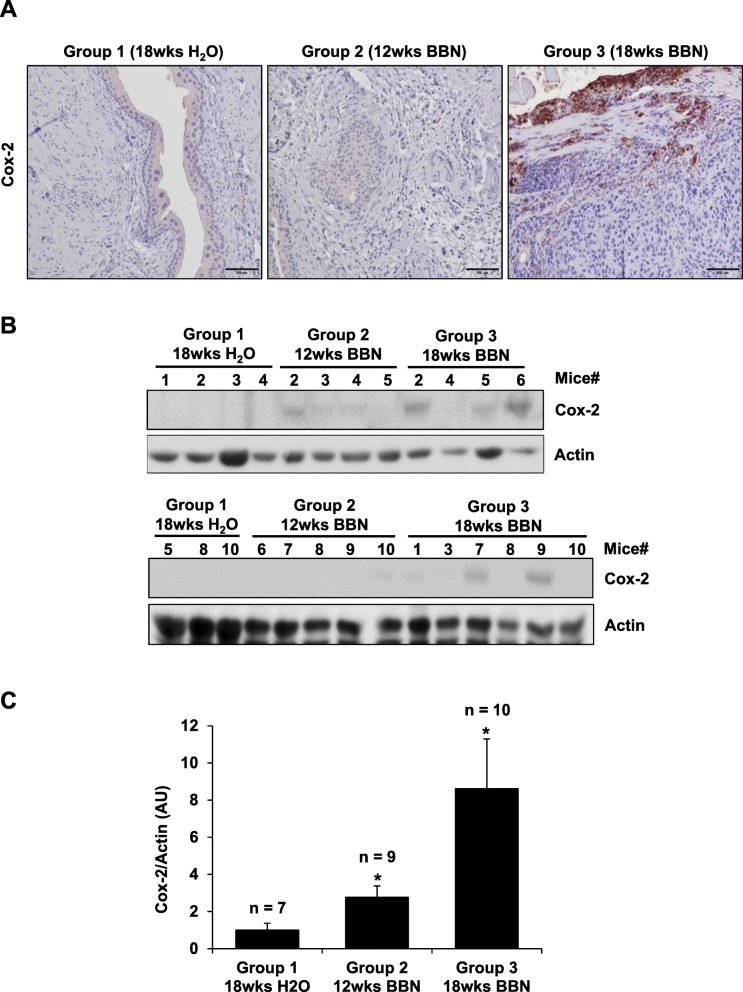
Upregulation of Cox-2 by BBN in bladder carcinoma. The upregulation of Cox-2 in BBN-induced bladder carcinoma was detected by (**a**) IHC, (**b**) WB, and (**c**) densitometry analysis of Cox-2/Actin protein bands from WB analysis using VisionWorks acquisition and analysis software (UVP). Bladder carcinoma in mice from Group 3 – 18wks BBN (*n* = 10) had 9-fold higher Cox-2 expression when compared to normal urothelium in mice from Group 1 – 18wks H_2_O (*n* = 7; control) and 3-fold higher in bladders with hyperplasia/CIS in mice from Group 2 – 12wks BBN (*n* = 9). Images taken by Leitz DMRB microscope, scale bar 100 μm. Actin was used as a WB loading control. **C** Data shown mean ± S.E. of the normalized Cox-2/Actin protein bands from WB analysis values of bladder from mice per each group determined by histological validation. Paired Student’s *t*-test was used to compare the upregulation of Cox-2 expression in BBN-exposed (Group 2 – 12wks BBN and Group 3 – 18wks BBN) with control group (Group 1 – 18wks H_2_O) (*p < 0.05)

The upregulation of Cox-2 in BBN-induced bladder cancer was detected by IHC and WB analysis (Fig. 4a-c). Bladder carcinoma lesions in mice from Group 3 (*n* = 10; 18wks BBN) had significantly higher Cox-2 expression when compared to normal urothelium in mice from Group 1 (*n* = 7; control) and bladder inflammation and hyperplasia in mice from Group 2 – 12wks BBN (*n* = 9; 12wks BBN). This result was also confirmed by WB analysis of the dissected bladder tissues from mice per each treatment group. The bladder tissues from mice in Group 2 – 12wks BBN and Group 3 – 18wks BBN had higher Cox-2 expression when compared to the bladder tissue from control mice (Group 1 – 18wks H_2_O), where there was no detectable Cox-2 expression. Densitometry analysis of Cox-2 protein bands from WB analysis was performed using VisionWorks acquisition and analysis software (UVP, Fig. 4c). There is a significant 3- and 9-fold increase in Cox-2 expression for bladder tissue from mice in Group 2 – 12wks BBN and Group 3 – 18wks BBN, respectively, when compared to bladder tissue from mice in Group 1 – 18wks H_2_O (*p < 0.05).

**Fig. 5:**
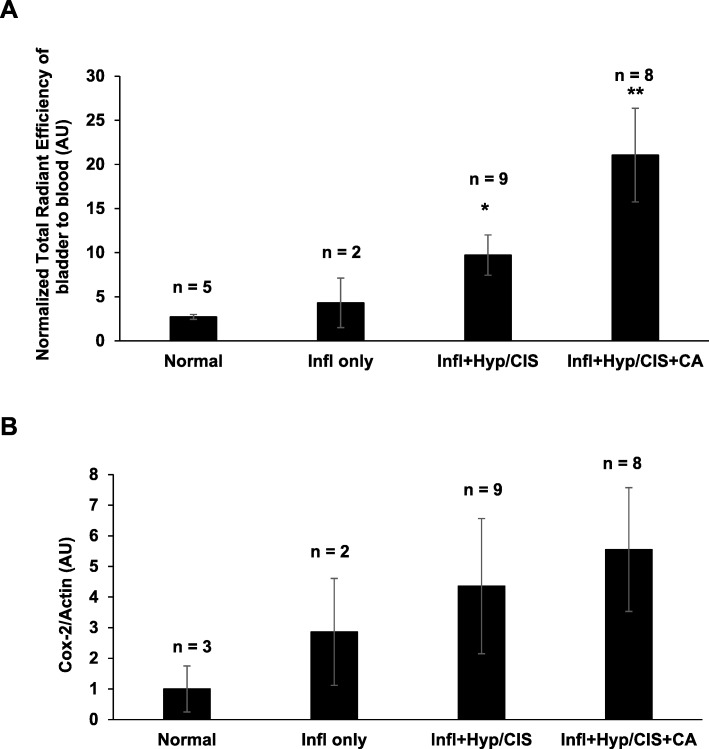
Correlation of fluorocoxib A uptake and Cox-2 with the progression of BBN-induced bladder carcinogenesis. **a** The normalized total radiant efficiency of fluorocoxib A uptake in bladder tissue determined by IVIS imaging system increased with the progression of bladder carcinogenesis determined by histological analysis in a BBN-induced bladder inflammation only (*n* = 2), bladders with CIS/Hyperplasia and inflammation (*n* = 9, *p < 0.05) and in bladders carcinoma lesions with CIS/Hyperplasia and inflammation (*n* = 8, **p < 0.01) as compared to normal bladders (*n* = 5). **b** Cox-2 expression determined by WB analysis increased with the progression of bladder carcinogenesis determined by histological analysis (**b**) in a BBN-induced bladder inflammation only (*n* = 2), bladders with inflammation and CIS/Hyperplasia (*n* = 9), and bladder carcinoma lesions with inflammation and CIS/Hyperplasia (*n* = 8) as compared to normal bladder (*n* = 3). Data show mean ± S.E. of the normalized total radiant efficiency values or Cox-2/Actin values of bladder from mice per each histologically determined group. Paired Student’s *t*-test was used to compare the upregulation of fluorocoxib A uptake or Cox-2 expression in BBN-exposed abnormal bladder tissue as compared normal bladders (*p < 0.05 and **p < 0.01)

The normalized total radiant efficiency of fluorocoxib A uptake in bladder tissue determined by IVIS imaging system increased with the progression of bladder carcinogenesis determined by histological analysis (Fig. 5a) in a BBN-induced bladder inflammation only (*n* = 2), bladder CIS/Hyperplasia with inflammation (*n* = 9, *p < 0.05), and in bladder with carcinoma lesions that also had lesions with CIS/Hyperplasia and inflammation (*n* = 8, **p < 0.01) as compared to normal bladder (*n* = 5). In addition, Cox-2 expression determined by WB analysis increased with the progression of bladder carcinogenesis determined by histological analysis (Fig. 5b) in a BBN-induced bladder inflammation only (*n* = 2), bladders with inflammation and CIS/Hyperplasia lesions (*n* = 9), and bladders with carcinoma lesions with inflammation and CIS/Hyperplasia lesions (*n* = 8) as compared to normal bladders (*n* = 3).

## Discussion

Current treatment options for patients diagnosed with bladder cancer is dependent on the stage and grade of cancer. After initial presentation and in office cystoscopy that demonstrates a concerning bladder lesion, patients are taken to the operating room at which time diagnostic and therapeutic TURBT is performed. Pathology results determines the presence of NMIBC versus MIBC. Therefore, an accurate understanding of the patient’s pathology and staging are critical in the provision of patients prognostic implications [4]. Unfortunately, WLC can fail to demonstrate occult bladder lesions, including CIS that are at high risk for the recurrence and/or progression. Thus, the new diagnostic tools are needed to improve detection during cystoscopy in order to distinguish non-invasive from muscle-invasive disease. This study demonstrates the improved visualization of Cox-2-expressing, carcinogen-induced early and late stage bladder cancer in an immunocompetent mouse model in vivo using the novel optical imaging agent, fluorocoxib A.

Interestingly, in our study we observed that mice exposed to 0.05% BBN in drinking water had increased water consumption as compared to mice from control groups as shown in Fig. 1c. Nitrosamines after ingestion are metabolized by the liver into several metabolites and excreted from the body through urine. In the bladder, the metabolites of nitrosamines come in contact with the urothelium and initiate the carcinogenic process resulting in DNA damage and development of both the early (NIMBC) and late stages (MIBC) of bladder cancer in mice [41, 42]. Bladder cancer in mice develops relatively early after BBN exposure with early-stage tumors developing after 12 weeks of exposure and late-stage tumors presenting after 18 weeks of exposure.

Fluorescence cystoscopy allows the visualization of the accumulated fluorescent contrast agents in the cancerous cells. Previous studies indicate improved diagnostic outcomes for patients with NMIBC using a fluorescent cystoscopy than WLC [43, 44]. There are several limitations to this method including rapid photo-bleaching during the cystoscopy procedure and a high false-positive rate (up to 30%) [45, 46]. In addition, the currently used non-selective photosensitizing agents (e.g. 5-ALA) during fluorescent cystoscopy procedures detect inflammation [45, 47], which contributes to the increased false positive detection rates. These limitations drive the need for the synthesis and validation of novel, cancer-specific contrast imaging agents that will improve the detection of the neoplastic lesions in the bladder resulting in improved patient prognosis. Fluorocoxib A, which is a Cox-2-selective imaging agent, is a prime candidate for the detection of bladder cancer during cystoscopy procedures due to its high stability and non-toxic effects. Previous preclinical studies indicate that fluorocoxib A has demonstrated to be highly specific and selective for detection of Cox-2-expressing head and neck, bladder, non-melanoma skin, and colorectal cancers [31–34].

In this study, the significant 3- and 7-fold TNR increases in the uptake of fluorocoxib A were observed in bladders dissected from mice in Group 2 – 12wks BBN and Group 3 - 18wks BBN, respectively, when compared to bladder of mice from Group 1 – 18wks H_2_O (control) that also correlated with the progression of bladder cancer (Fig. 2). Moderate uptake of fluorocoxib A was observed in both the liver and muscle tissue as shown in Fig. 2c and d due to the metabolism and excretion route of fluorocoxib A [31]. These results demonstrate the specificity of fluorocoxib A uptake by the cancerous bladder tissue when compared to normal urothelium (Group 1 – 18wks H_2_O) where no fluorocoxib A uptake was detected. Furthermore, the range of fluorocoxib A signal between the groups indicates the ability of fluorocoxib A to detect different stages of bladder tumors, including hyperplasia and CIS vs carcinoma lesions that was confirmed by histology analysis (Fig. 3). All bladder tissue from mice in Group 3 (18wks BBN) displayed signs of inflammation and/or CIS/Hyperplasia in developed bladder carcinomas as shown in a Table 2 and Fig. 5. These results confirm the progression of bladder carcinogenesis induced by BBN exposure and incidence rates are consistent with previously published BBN studies [37, 38, 42]. Ki67 is a cellular marker associated with cell proliferation and often correlates with patient prognosis and aids in determining clinical course of treatment. The progression of bladder carcinogenesis was confirmed by the presence of increased expression of Ki67 positive cells in bladder urothelium from mice treated with BBN (Group 2 – 12wks BBN and Group 3 – 18wks BBN) (Fig. 3). Uroplakin-1A (UP1a) is a member of a group of cell-surface proteins and is highly expressed in normal bladder urothelium. The downregulation of UP1a indicated a loss of normal urothelium and changes in morphology of the bladder tissues as the cancer progressed as was also confirmed in our study (Fig. 3).

Cox-2 plays a key role in modulating cellular proliferation, apoptosis, and tumor invasion. The increased Cox-2 expression has also been reported to be correlated with tumor grade and poor clinical outcome for patients diagnosed with bladder cancer [27–30]. In our study we confirmed that developed bladder carcinomas in mice had significantly higher Cox-2 expression when compared to normal urothelium, bladder inflammation, and hyperplasia in bladders from BBN-treated mice (Fig. 4). There was a significant 3- and 9-fold increase in Cox-2 expression in bladder tissues in mice for Group 2 – 12wks BBN and Group 3 – 18wks BBN, respectively, when compared to Group 1 – 18wks H_2_O (Fig. 4c). These results also correlate with the increased uptake of fluorocoxib A and Cox-2 expression in the bladders of mice that were positive for CIS/Hyperplasia with inflammation and carcinomas with CIS/Hyp and inflammation (Fig. 5). The detected COX-2 in bladder cancer tissue by fluorocoxib A could be used as a biomarker not only for the detection of bladder cancer, but also as a prognostic factor for poor clinical outcome of patients diagnosed with COX-2-expressing bladder cancers.

## Conclusions

The specific and increased fluorocoxib A uptake in the BBN-induced bladder carcinomas in mice in vivo correlated with the progression of bladder carcinogenesis and with increased Cox-2 expression. Currently, conventional imaging technologies for the detection of bladder cancer have several limitations, primarily lacking the ability to detect early stage bladder cancer and poor visualization of tumor margins during resection procedures. The development of novel techniques and imaging agents, such as fluorocoxib A, could potentially greatly improve patient prognosis by aiding in both the diagnostic and treatment procedures.

## Data Availability

All data generated or analyzed during this study are included in this published article.

## Abbreviations

5-ALA: 5-aminolevulinic acid
BBN: N-butyl-N-4-hydroxybutyl nitrosamine
BLC: Blue light cystoscopy
CIS: Carcinoma-in-situ
CLE: Confocal laser endomicroscopy
Cox-2: Cyclooxygenase-2
Fluorocoxib A: a *N*-[(5-carboxy-X-rhodaminyl) but-4-yl]-2-[1-(4-chlorobenzoyl)-5-methoxy-2-methyl-1*H*-indol-3-yl] acetamide
Hyp: Hyperplasia
IHC: Immunohistochemistry
NBI: Narrow band imaging
NMIBC: Non-muscle invasive bladder papillary carcinoma
OCT: Optical coherence tomography
PDD: Photodynamic diagnosis
TCC: Transitional cell carcinoma
TNR: Tumor-to-noise ratio
TURBT: Transurethral resection of bladder tumor
UP1a: Uroplakin-1A
WB: Western blotting
wks: Weeks
WLC: White light cystoscopy
